# Socio-psychological predictors of school dropout intention among Italian adolescents: evidence from a large-scale study

**DOI:** 10.3389/fpsyg.2026.1705896

**Published:** 2026-06-01

**Authors:** Federica Ruzzante, Riccardo Loconte, Marcella Romeo, Gianluca Sesso, Sonia D’Arcangelo, Francesca Maggi, Emiliano Ricciardi, Pietro Pietrini

**Affiliations:** 1 MoMiLab Research Unit, IMT School for Advanced Studies Lucca, Lucca, Italy; 2 Developmental Psychiatry and Psychopharmacology Unit, IRCCS Stella Maris Foundation, Pisa, Italy; 3 Neuroscience Lab, Intesa Sanpaolo Innovation Center S.p.A., Torino, Italy

**Keywords:** adolescence, environmental factors, individual factors, mental health, psychopathology, school dropout

## Abstract

**Introduction:**

School dropout is the premature interruption or extended completion of education, posing significant challenges for both individuals and educational systems worldwide.

**Methods:**

This study explores how psychosocial factors influence the intention to dropout of school among 2,646 students from 13 secondary schools in Italy. By assessing individual (e.g., personality, behavior, mental health) and environmental (e.g., family, school context) factors, the study seeks to understand the role of psychological distress in dropout intentions. Specifically, individual factors were measured through the Youth Self-Report, Bergen Social Media Addiction Scale, Internet Gaming Disorder Scale, Eating Attitude Test, Hikikomori Questionnaire, and Big Five Inventory-10. Environmental factors were measured using the Word Health Organization Quality of Life, Parental Bonding Inventory, and the Questionnaire de dépistage d’élèves à risque de décrochage scolaire. Socio-demographic factors and social desirability bias (using the Impression Management scale from the Balanced Inventory of Desirability Responding) were also measured.

**Results:**

Multiple linear regression analyses, controlling for gender, socioeconomic status, age, and social desirability, revealed family supervision as a crucial protective factor against dropout. Conversely, attention-related issues such as impulsivity and difficulty concentrating were strong predictors of dropout intentions, indicating their detrimental effect on academic performance and study habits.

**Discussion:**

These findings highlight the importance of family involvement and mental health support to reduce dropout rates. Addressing both environmental and psychological factors is critical for promoting student retention and academic success.

## 1 Introduction

School dropout represents a significant challenge for educational systems worldwide (European Commission, 2013). It is defined as the early interruption and definitive abandonment of the educational path or the achievement of the level of education in a significantly longer time than expected, according to the study program (European Commission, 2013). This phenomenon not only affects students individually but also has broader socio-political and economic consequences (Rumberger, 2012). Although a certain dropout level may be inevitable due to family circumstances, economic factors, or personal choices, higher dropout rates may reflect systemic problems within educational institutions and broader social disparities (Rumberger, 2012). The present study aims to jointly examine the factors associated with school dropout and uncover which are at the root of this issue.

Within the school dropout framework, extensive literature has identified key contributing factors. For instance, socioeconomic status was defined as a critical determinant of the phenomenon, since students coming from low-income families are more likely to abandon school beforehand. Specifically, family income, parental education level, and access to educational resources significantly affect educational attainment (Rumberger, 2012). Relatedly, family dynamics, such as family structure and parental involvement, also play a crucial role, since students from single-parent families, or whose parents are less involved in the educational pathway, are at higher risk of dropping out. However, emotional support and academic encouragement from parents can reduce such risk (McNeal, 1999; Pong and Ju, 2000). Also, school environment factors such as teacher support, school safety, and academic quality can either increase or decrease dropout risk (Lee and Burkam, 2003; Rumberger and Thomas, 2000). Similarly, community environment, peer influence, and employment opportunities (i.e., external factors) have this dual valence in dropout risk. Indeed, students living in communities with higher criminality or those influenced by peers who have abandoned their education pathway are more likely to prematurely leave the school (Ensminger et al., 1996; Entwisle et al., 2004). Finally, individual characteristics, such as behavioral patterns, attitudes towards school, and academic performance when negatively denoted, play a negative role and increase the likelihood of dropping out (Alexander et al., 2001; Jimerson et al., 2000). Among them, mental health issues represent a key factor in school dropout. Indeed, most psychopathological issues, such as depression, anxiety, personality disorders, and behavioral disorders, have their onset during adolescence (Jones, 2013). Students exhibiting depressive symptoms had a significantly higher probability of disengaging from school activities and reported poor academic results, leading to higher dropout rates (Esch et al., 2014). Moreover, mental health problems may impair cognitive functions, including memory, decision-making, and concentration, thus increasing known risk factors such as grade repetition and low achievements (Kessler et al., 1997). Other studies focusing on attention and hyperactivity issues described how a diagnosis of attention deficit and hyperactivity disorder (ADHD) negatively impacts school environments, since individuals are more likely to engage in conflicts with peers and teachers and to disengage from the educational system (Barkley, 2015). Additionally, stigma related to mental health issues may prevent help-seeking, further exacerbating school difficulties (Corrigan et al., 2003): previous studies reported that 10–20% of individuals experience mental health problems during adolescence, and the European Commission identified these as primary factors influencing early school leaving (European Commission, 2013).

While dropout rates are decreasing in Europe, Italy remains above the European average. Indeed, the Italian dropout rate is 13.1%, (individuals aged 18–24 who left education and training without obtaining an upper secondary qualification) compared to a European rate of 10% (European Commission, 2023). Crucially, despite the decrease of Italian school dropout over the last few years, regional disparities remain significant, with Southern regions reporting higher dropout rates compared to Northern regions (Bratti et al., 2007; Cappellari and Lucifora, 2009). In this framework, multilevel Italian studies have shown socio-economic inequalities are linked to psychosocial distress. For instance, higher income inequalities at both municipal and regional levels correlates with greater risk of developing anxiety and depressive symptoms, mostly in the southern regions (Rossi et al., 2024). These regional inequalities also limit access to mental health services, a situation that has been worsened by the COVID-19 pandemic (Petrelli et al., 2024). Further, longitudinal research involving a region of Center Italy (i.e., Tuscany) highlighted that adolescents who live in areas with greater economic disparities experience increasing psychological symptoms over time (Lipari et al., 2023). Previous studies in Italy confirm that socioeconomic status (Barone, 2006; Checchi and Flabbi, 2006), family background (Azzolini and Barone, 2013), school environment (Agasisti and Cordero-Ferrera, 2013; Frattini and Meschi, 2019), regional disparities (Bratti et al., 2007; Cappellari and Lucifora, 2009), and individual characteristics (Checchi et al., 2013; Sacco and Le Rose, 2022) play a role in the ability of students to complete their educational path. Great attention has been given to the role of socio-economic factors and their interconnection in school dropout. Cultural attitudes towards education can influence completion rates and are linked to regional disparities, also reinforcing gender inequalities. Moreover, family income (Checchi and Flabbi, 2006), parental employment status (Ballarino et al., 2009) and education levels (Brunello and Checchi, 2007), among the socio-economic factors, are crucial predictors of dropout in Italy. Overall, these findings highlight a persistent critical situation. Consistently, financial difficulties and psychological distress were recently associated with reduced academic performance among Italian university students during COVID-19 (Campodifiori et al., 2010).

Previous studies on the Italian situation focused mainly either on socio-economic factors or on other individual factors alone. At the same time, psychological factors and the impact that psychopathological issues may have on school dropout are understudied. Thus, the present study aimed to investigate from a broader point of view which factors exert a protective role or mainly contribute to school dropout within the Italian education system, by specifically addressing psychosocial factors, mainly neglected in previous studies. In particular, we pursued our aim by enrolling a large sample of students from secondary school and administering a survey composed of validated questionnaires and scales to assess individual (i.e., psychological and behavioral factors, physical health, and quality of life) and environmental factors (i.e., school, family, and socio-economic factors), thus allowing us to quantitatively estimate the correlations between each factor and the intention to school dropout. It is worth noting that, given the cross-sectional design of the present study, our outcome measure refers to students’ dropout intention, rather than actual dropout behavior.

## 2 Materials and methods

This study employed a descriptive, cross-sectional, quantitative survey design to investigate the correlations between school dropout intentions and individual and environmental factors within a large sample of Italian adolescents from secondary school.

### 2.1 Participants

A total of 3,946 students were recruited from 13 educational institutions in two Italian cities, Turin and Lucca. These two cities were chosen to include different urban realities, specifically, a large industrial metropolis in northern Italy, Turin, with a socio-economic context characterized by significant cultural diversity and a wide range of educational and professional opportunities, and a smaller city in the Tuscany region, Lucca, with a relatively more homogeneous socio-economic context. The educational institutions were preselected based on their territorial representativeness regarding the type (high schools vs. technical institutes vs. vocational institutes) and the socio-economic status of the neighborhood in which the institution was located. Private and parochial institutions were excluded to maintain the focus on public schools. The recruitment of the educational institutes was conducted by contacting the school principals through the help of Fondazione Links and Fondazione per la Scuola (i.e., foundations that serve as instrumental bodies of Fondazione Compagnia di Sanpaolo to connect public and private actors within the education community). In Turin, nine educational institutions participated in the present study, with a total of 2,458 students (62% of the sample) enrolled. In Lucca, four educational institutions were involved, with a total of 1,488 students (38% of the sample) enrolled.

Three thousand seventy-four students (78% of the sample) provided the mandatory informed consent (and assent) to access the survey; 428 participants (14% of respondents) were excluded from further analysis due to incomplete responses or excessive time taken to complete the survey. Thus, a total of 2,646 valid responses were collected, comprising 79% from the Turin subsample and 21% from the Lucca subsample. The responding sample was well balanced for gender, with a slight predominance of females (50.93%) over males (45.67%). Additionally, 1.36% of participants referred to their gender as “Other,” and 1.96% preferred not to specify their gender. In terms of age distribution, the sample was mainly concentrated within the 14–18 age group (M_age_ = 16.46, SD_age_ = 1.60). The sample was also balanced for class, with 21% of students in their first year of school, 20% in their second year, 19% in third year, 20% in fourth year, and 20% in fifth year. The sample was mainly composed of students whose mother tongue was Italian (88%), with approximately 12% of students who were non-native speakers of Italian.

### 2.2 Procedure

The battery of questionnaires was computerized in an online survey via Qualtrics. The survey comprised 294 items, with an initial section dedicated to socio-demographic data and a second section including the questionnaires (see Section 2.3). To avoid any potential order confounder, the questionnaires and the items within each questionnaire were randomized. The survey required 30–40 min to be completed. Each school independently managed the administration of the survey, with most schools administering it in the classroom during a predetermined school hour, supervised by one or more teachers. Students unable to complete the survey at school could complete it at home or later in the classroom.

### 2.3 Measures

**Figure 1:**
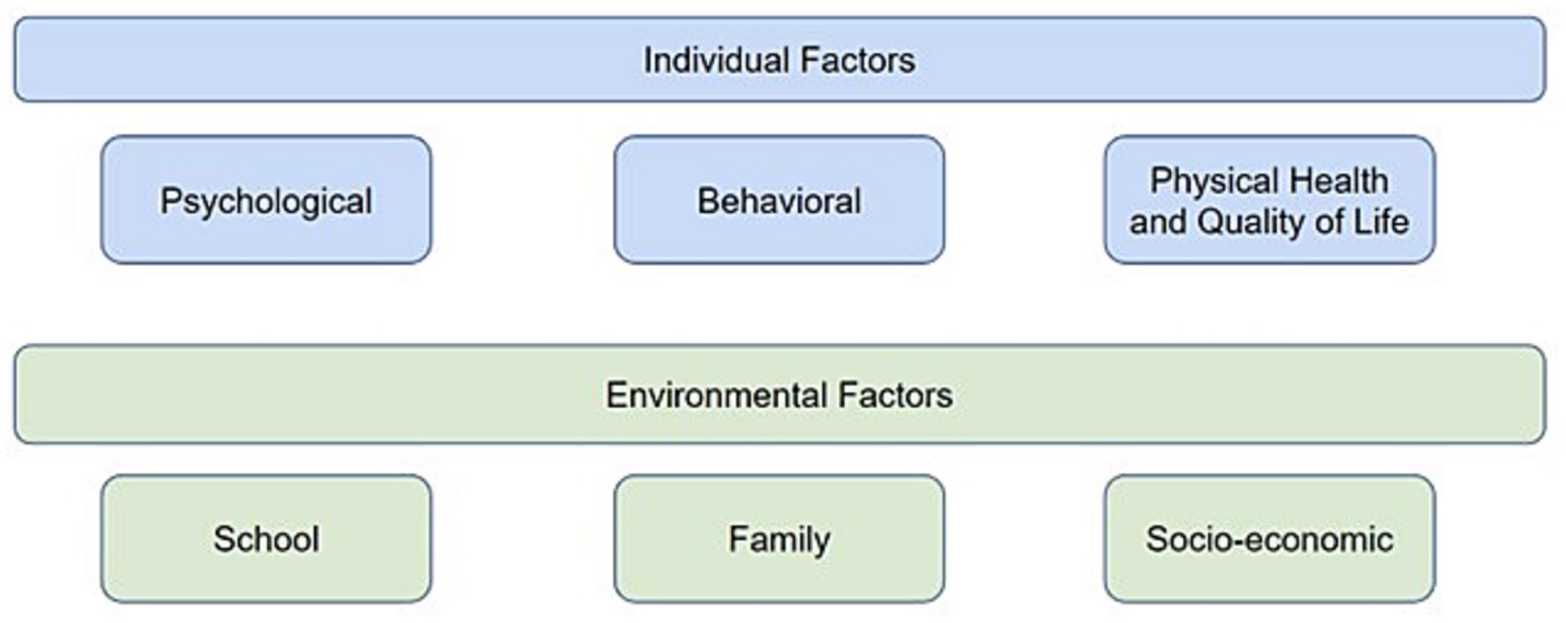
Factors identified from the literature review as strongly associated with school dropout. Psychological and behavioral factors, along with physical health and quality of life, were conceptualized as individual factors. In contrast, school, family, and socioeconomic factors were conceptualized as environmental factors.

From a literature review, six main factors were identified as the most associated with dropout intentions: three individual factors (psychological, behavioral, and quality of life) and three environmental factors (school, family, socioeconomic status). These are presented in Figure 1. For each of these six factors, at least one reference questionnaire that met the following inclusion criteria was selected from the scientific literature:

1. The questionnaire was validated in an adolescent population;
2. The questionnaire was employed in scientific studies investigating school dropout;
3. The questionnaire was validated in Italian.

The battery of selected questionnaires comprised 294 items (Table 1).

**Table 1:** Overview of factors examined, corresponding questionnaires, and the number of items within each questionnaire.

Factors (% of items)	Questionnaire	Num. of items
Psychological and behavioral (67%)	Youth Self Report (YSR)	112
	Bergen Social Media Addiction Scale (BSDMAS)	6
	Internet Gaming Disorder Scale-Short Form (IGDS9-SF)	9
	Eating Attitude Test (EAT-26)	26
	Hikikomori Questionnaire (HQ-25)	25
	Impression Management scale (IM)	8
	Big Five Inventory-10 (BFI-10)	10
Physical Health and Quality of life (9%)	World Health Organization Quality of Life (WHOQoL)	26
School (11%)	Questionnaire du dèvaluation du dècrochage en milieu scolaire (DEMS)	33
Family (9%)	Parental Bonding Inventory (PBI)	25
Socio-demographic factors (4%)	–	14

Percentages in parentheses indicate the proportion of total items dedicated to each factor, aggregated from one or more pertinent questionnaires.

#### 2.3.1 Socio-demographic factors

Demographic factors such as gender, age, type of institution attended, and city of residence were collected prior to administering the survey. The socio-economic-cultural status (SECS) of students was estimated by employing several proxy variables derived from the INVALSI Student’s Questionnaire (Agasisti and Cordero-Ferrera, 2013; Sacco and Le Rose, 2022). Those variables related to (1) parents’ birthplace, (2a) the availability of a quiet place to study, (2b) a computer for study purposes, (2c) a desk for doing homework, (2d) encyclopedias, (2e) internet access for research, a (2f) security alarm, (2g) a private room for the student, (3a) the number of bathrooms and (3b) cars in the household, and (4) the number of books at home, excluding schoolbooks.

#### 2.3.2 Youth Self-Report

Youth Self-Report (YSR; 32) is a primary screening instrument for emotional and behavioral problems that has been widely used in both the general population and in clinical settings. The instrument exhibits high reliability (Cronbach’s *α* > 0.80) and excellent consensus validity. The YSR comprises 112 items, divided into eight specific subscales and three general subscales. The eight specific subscales refer to (1) anxiety problems, (2) affective problems, (3) social withdrawal, (4) somatic problems, (5) social problems, (6) attentional problems, (7) disruptive behaviors, and (8) aggressive behaviors. General subscales measure the presence of internalizing problems, externalizing problems, and total problems. For this study, the Italian version of the YSR validated by Frigerio and Montirosso (2002) was administered to participants, and each item was rated on a three-point scale (0 = not true; 1 = sometimes true; 2 = mostly true).

#### 2.3.3 Bergen Social Media Addiction Scale

Bergen Social Media Addiction Scale (BSMAS; (Andreassen et al., 2016)) is a self-report questionnaire for assessing social media addiction. It consists of 6 items that measure key symptoms of social media addiction, such as salience, mood modification, tolerance, withdrawal, conflict, and relapse. Each item pertains to experiences within the past year and is answered on a 5-point scale (1 = very rarely; 5 = very often). Higher scores on the BSMAS are indicative of a higher risk for social media addiction. The Italian version of the BSMAS, developed by Monacis et al., (2017), validated through confirmatory factor analysis (CFA), showed excellent fit indices and strong internal consistency (Cronbach’s *α* = 0.88). In its validation study, measurement invariance was also established across gender and age groups, ensuring the scale’s reliability and validity for diverse populations.

#### 2.3.4 Internet Gaming Disorder Scale

Internet Gaming Disorder Scale—Short Form (IGDS9-SF; Pontes and Griffiths, 2015) is a self-report questionnaire for assessing the severity and negative effects of Internet Gaming Disorder (IGD) by examining online and/or offline gaming activities occurring over a 12-month period. The scale consists of nine items corresponding to the nine diagnostic criteria proposed by the DSM-5, which lists Internet Gaming Disorder as a condition requiring further study, with responses on a 5-point scale (1 = never, 5 = very often). Higher scores indicate a higher degree of IGD. The instrument, in its Italian validation developed by Monacis et al., (2016), has excellent reliability (Cronbach’s *α* = 0.96).

#### 2.3.5 Eating Attitude Test

Eating Attitude Test (EAT-26; Garner and Garfinkel, 1979) is a widely used self-assessment instrument composed of 26 questions that measure symptoms and concerns typical of eating disorders, including dietary restrictions, bulimia, and preoccupation with food and weight. Each item was scored on a 6-point scale (1 = never, 6 = always). Higher total scores are indicative of an elevated risk for the development of eating disorders. A total score exceeding 20 is intended as an indication that the individual necessitates to undergo further assessment by a mental health professional. The instrument, in its Italian validation (Dotti and Lazzari, 1998; Ruggiero et al., 1999), shows very good reliability (Cronbach’s *α* > 0.80) and can identify cases at risk for eating disorders. The EAT-26 is one of the most widely used tools for research and clinical practice on eating disorders, especially within school and sports settings.

#### 2.3.6 Hikikomori Questionnaire

Hikikomori Questionnaire (HQ; Teo et al., 2018) is an assessment tool developed to identify cases of prolonged social withdrawal, a phenomenon relatively frequent in Japan and known as hikikomori. The questionnaire consists of 25 items that measure three key dimensions of hikikomori: (reduced) socialization, isolation, and (lack of) emotional support. Each item is rated on a 5-point scale (0 = strongly disagree; 4 = strongly agree), with total scores ranging from 0 to 100. The Italian validation of the HQ (Amendola et al., 2022; Fino et al., 2023) shows good internal reliability and satisfactory convergent validity with other social withdrawal assessment instruments.

#### 2.3.7 Big Five Inventory-10

Big Five Inventory-10 (BFI-10; Rammstedt and John, 2007) is a measurement scale consisting of 10 items to assess the five main dimensions of personality according to the Big Five Factor Model (Big Five): Extroversion, Agreeableness, Conscientiousness, Emotional Stability, Openness to Experience. Each item is rated on a 5-point scale (1 = strongly disagree; 5 = strongly agree). Confirmatory factor analysis showed adequate 5-factor structure and good fit indices for the Italian version of the BFI-10, which was developed by Guido et al., (2015). The subscales, consisting of 2 items each, showed acceptable levels of internal consistency and good convergent validity with other personality measures. The BFI-10 has been proposed as a short but valid instrument for assessing personality dimensions according to the Big Five model in research settings where time-saving is a priority.

#### 2.3.8 Impression Management

With the aim of identifying and adjusting for responses that may be influenced by social desirability, Impression Management (IM) scale, derived from the Balanced Inventory of Desirable Responding questionnaire (BIDR; Paulhus, 1991), was administered to participants. The IM scale assesses the intentional and conscious presentation of a favorable public image and consists of 8 items rated on a 6-point scale (1 = strongly disagree; 6 = strongly agree). In its Italian validation, developed by Bobbio and Manganelli (2011), the IM scale shows good reliability (Cronbach’s *α* = 0.73) for administration in online validation samples.

#### 2.3.9 World Health Organization Quality of Life

World Health Organization Quality of Life (WHOQoL; De Girolamo et al., 2000a,b) is a self-assessment tool for quality of life developed by the World Health Organization (WHO). It was designed as a generic validated instrument to assess perceived quality of life in clinical settings, reflecting the holistic approach to health promoted by WHO. The WHOQoL includes 26 items that investigate four main areas: physical health, psychological health, social relationships, and quality of the environment. Additionally, it includes two general items on the overall perceived quality of life and health. This abbreviated version of the original 100-item WHOQOL was created by selecting the most representative items for each of the 24 sections, and validated on the Italian population by De Girolamo et al. (2000a,b). The WHOQOL showed good psychometric properties, including internal consistency (Cronbach’s *α* from 0.76 to 0.93), concurrent validity, and test–retest stability.

#### 2.3.10 Student at-Risk of Dropping out Screening Questionnaire

The Student at Risk of Dropping out Questionnaire (Questionnaire de dépistage d’élèves à risque de décrochage scolaire; DEMS; Potvin et al., 2004) is a self-assessment instrument consisting of 33 questions, originally developed and validated to identify indirect indicators of school dropout in secondary school students from Quebec (Potvin et al., 2004). Those indicators refer to (i) parental involvement and (ii) parental supervision, (iii) attitude toward school, (iv) perception of school performance level, and (v) school aspirations. In its original version, the DEMS demonstrated robust reliability and validity, as well as good predictive validity, with the total score predicting 80.3% of students in the dropout group and categorizing them into three levels of dropout risk (low vs. moderate vs. severe). This questionnaire was the only one that has not already been translated in the Italian language. Therefore, the original questionnaire was first translated into Italian by the primary author and then back-translated by an independent translator. The back-translation was finally compared to the original to assess the validity of the translation. The Italian translation demonstrated good internal validity (Cronbach’s *α* = 0.85).

#### 2.3.11 Parental Bonding Inventory

Parental Bonding Inventory (PBI; Parker et al., 1979) is a questionnaire assessing parenting style as perceived by children and adolescents, measuring the dimensions of care and protection and providing a basis for identifying four patterns of parenting behavior: absentee parenting, optimal parenting, affectionless control, and affective control. The PBI consisted of 25 items scored on a four-point scale (0 = unlikely; 3 = very likely). In its Italian validation developed by Scinto et al. (1999), the PBI showed good psychometric properties in terms of validity and reliability (Cronbach’s *α* > 0.80).

#### 2.3.12 School dropout intention

Dropout intention (DOI) was measured using ten items designed to capture the individual negative attitudes toward school and their intrinsic and extrinsic motivations to pursue studies. Specifically, the first five items investigate the subject amotivation about continuing their education. Those items were reformulated from the amotivation subscale of the Academic Motivation Scale, developed by Alivernini and Lucidi (2008). Examples of items are “Once I had good reasons to go to school; however, now I wonder if I should continue” and “*Sometimes I am not sure if I want to continue studyin*g.” The second five items start with the sentence “*The reason why I go to school is…”* and investigate the underlying reasons for attending school, focusing on students’ intrinsic and extrinsic motivations to pursue studies. Examples of items were “*because education is important to me*” and “*because I have to.*” DOI items are rated using a 5-point scale (1 = completely disagree; 5 = completely agree). In this study, the internal consistency of the ten items measuring DOI was high (Cronbach’s *α* = 0.83), ensuring the reliability of the measure.

### 2.4 Ethics statement

The study was in accordance with the Declaration of Helsinki. The protocol was approved by the Joint Ethical Committee for Research of Scuola Normale Superiore and Scuola Superiore Sant’Anna (Protocol Number: 32/2022). Participation was voluntary, with written informed consent obtained for students aged over 18 years and from parents or legal guardians for students aged under 18 years. At the time they received the survey, participants over 18 directly provided their consent, while those under 18 both obtained consent from their parents or guardians and provided their assent. Notably, participants under 18 years of age could obtain consent from their parents or legal guardians but still decide not to provide their assent at the time of the survey completion and thus would not take part in the study. Participants were informed that they could withdraw their consent at any time during or after participating in the study. All survey responses were collected anonymously, and no personal data were recorded. Data was stored following the university’s data-management policy and managed in accordance with the European General Data Protection Regulation and the Italian Legislative Decree no. 196/2003, meaning that only authorised researchers had access to it.

### 2.5 Analysis plan

Data analyses were performed in RStudio (RStudio Team, 2020). As a first step, total scores for each questionnaire were computed by summing the scores provided for each item, following the procedures described in their validation papers. This included reversing items where necessary to maintain the validity and reliability of the measures. Descriptive analysis was then performed to investigate the prevalence of psychological distress in our sample as measured through the administered questionnaires. Secondly, differences in dropout intention scores were analyzed regarding gender, age, type of institution, class (first year vs. fifth year), recruitment city (Turin vs. Lucca), and SECS. Finally, to investigate the factors associated with dropout intention, we adopted a multivariate modeling approach. Specifically, a single multiple linear regression model was estimated including all predictors of interest simultaneously, while controlling for age, gender, socioeconomic status (SECS), and impression management. This approach allows for the estimation of the unique contribution of each predictor while accounting for shared variance among variables, thereby reducing the risk of inflated Type I error associated with multiple separate models. Multicollinearity was formally evaluated using Variance Inflation Factors (VIFs), with values below conventional thresholds indicating acceptable levels of collinearity (see Supplementary materials).

To facilitate interpretation across variables measured on different scales, standardized regression coefficients (*β*) were computed by refitting the model on standardized variables. Model fit was assessed using *R*^2^ and adjusted *R*^2^ indices. Additionally, a nested model comparison was conducted by contrasting the full model with a reduced model including only covariates, using an analysis of variance (ANOVA), to evaluate the incremental explanatory power of the psychological and contextual predictors. All analyses were conducted in Rstudio.

## 3 Results

### 3.1 Descriptive analyses

**Figure 2:**
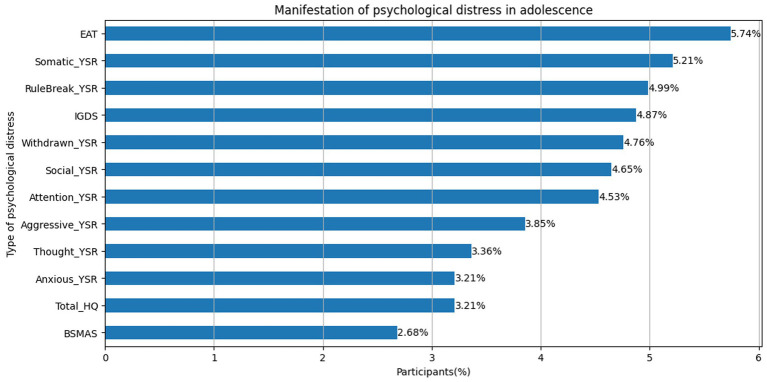
Prevalence of different manifestations of psychological distress in adolescence. Labels containing the YSR acronym refer to psychological problem scales derived from the Youth Self-Report questionnaire. The x-axis represents the percentage of participants scoring more than two standard deviations above the sample mean, while the y-axis lists the psychological dimensions assessed.

An initial descriptive analysis was performed to assess the prevalence of psychological distress in our sample. To this aim, final scores for the measured variables were first computed and then standardized. Figure 2 illustrates the prevalence of vulnerability index scores, where a score is considered significant if it is more than two standard deviations from the mean (Figure 2). Particularly, as shown in Figure 2, it can be observed that each of the psychological and/or behavioral distress manifestations was found in 2–5% of the surveyed population. Among the explored variables of interest, eating problems, somatic complaints, rule-breaking behaviors and gaming issues were the most prevalent in our sample (see Figure 2). Additionally, out of a total of 2,647 respondents, 635 (23.99%) showed a risk score in at least one of the psychological distress factors, such as social media addiction, social withdrawal, or norm-violating behaviors. Among these at-risk subjects, 286 (10.80%) showed the concomitant presence of two or more factors.

### 3.2 The role of sociodemographic variables in school dropout

We then compared dropout intention scores according to categorical demographic data such as gender, type of institution, class, and recruitment city, by means of a series of ANOVAs after assumptions were confirmed (Shapiro–Wilk test for normality, Levene’s test for homogeneity of variances). A first one-way ANOVA revealed a significant main effect of gender on dropout intention, *F*(3, 2,643) = 7.72, *p* < 0.001. Post-hoc tests indicated that this effect was primarily driven by a significant difference between male and female students, with male students reporting significantly higher dropout intention (Mean difference = −1.01, SE = 0.31, *p* = 0.007). While individuals who preferred not to indicate their gender showed a significantly higher intention to dropout than girls (*F* = 7.17, *ƞ*^2^ = 0.0086, *p* = 0.0309). Students from Technical and Vocational Institutes were at higher risk compared to those from High Schools (*F* = 15.11, *ƞ*^2^ = 0.0113, *p* < 0.0001). Furthermore, an increasing trend in dropout rates, though non-significant, was found among students from first to fifth classes (*p* = 0.0694). As for the recruitment sites, we did not find significant differences in dropout rates between Turin and Lucca (*p* = 0.8743). Finally, for continuous demographic data, we computed Pearson’s correlation coefficients: the intention to dropout was significantly and negatively correlated with SECS, showing a small effect (*r* = −0.12; *p* < 0.001). Additionally, age was significantly and positively associated to dropout intentions (*r* = 0.07; *p* < 0.001), albeit with a very small correlation and possibly influenced by the large sample size.

### 3.3 Regression analyses

A multiple linear regression model including all predictors and covariates was conducted to examine factors associated with dropout intention. The full model explained a substantial proportion of variance in dropout intention scores (*R*^2^ = 0.469, adjusted *R*^2^ = 0.463), and significantly improved model fit compared to the covariate-only model (Δ*F*_(26, 2,614)_ = 63.24, *p* < 0.001), indicating that the inclusion of psychological, behavioral, and social variables provided meaningful explanatory power. Importantly, multicollinearity diagnostics indicated acceptable levels of collinearity across predictors, with all VIF values below 4, suggesting that the estimates were stable and not unduly biased by overlapping constructs. This addresses concerns regarding inflated coefficients observed in earlier analyses.

Standardized regression coefficients (*β*) were examined to facilitate interpretation. Among the psychological and behavioral factors, the strongest positive associations with dropout intention were found with the total score of the Hikikomori Questionnaire (*β* = 0.08, *p* = 0.002), and the Attention Problems (*β* = 0.07, p = 0.002) and Somatic Complaints subascales (*β* = 0.06, *p* = 0.004) of the YSR. School factors also played an important role with the Attitudes subscale of the DEMS emerging as the most robust predictor (*β* = 0.51, *p* < 0.001), followed by the Aspiration subscale (*β* = 0.07, *p* < 0.001), and the Self-Efficacy subscale (*β* = 0.04, *p* = 0.031). Additional significant positive predictors included the role of family care, as emerged from the Care subscale of the PBI (*β* = 0.06, *p* = 0.009).

Conversely, several variables were negatively associated with dropout intention. These included Aggressive Behavior (*β* = −0.06, *p* = 0.017) and Thought Problems (*β* = −0.06, *p* = 0.029) subscales of the YSR, from the psychological and behavioral factors, the role of Quality of Life (*β* = −0.04, *p* = 0.043), and the parental overprotection (PBI) (*β* = −0.05, *p* = 0.006) (see Table 2).

**Table 2:** Results of the regression analyses.

Factors	Variables	St. Beta (95% CI)	St. Err.	*t*-value	*p*-value
Psychological and behavioral	**YSR—Attention Problems**	**0.07 (0.03, 0.12)**	**0.02**	**3.08**	**0.002** ^**^
	YSR—Rule Breaking Behaviors	0.03 (−0.02, 0.07)	0.02	1.07	0.286
	**YSR—Aggressive Behaviors**	**−0.06 (−0.11, −0.01)**	**0.03**	**−2.38**	**0.017** ^*^
	YSR—Withdrawn/Depressed	0.03 (−0.03, 0.08)	0.03	0.93	0.353
	YSR—Anxious/Depressed	0.03 (−0.02, 0.09)	0.03	1.19	0.23
	**YSR—Thought Problems**	**−0.06 (−0.11, −0.1)**	**0.03**	**−2.19**	**0.029** ^*^
	YSR—Social Problems	−0.009 (−0.06, 0.04)	0.03	−0.35	0.726
	**YSR—Somatic Complaints**	**0.06 (0.02, 0.10)**	**0.02**	**2.88**	**0.004** ^**^
	YSR—other problems	−0.02 (−0.06, 0.02)	0.02	−0.83	0.406
	BSMAS—total score	0.03 (0.00, 0.07)	0.02	1.85	0.064
	IGDS—total score	0.004 (−0.03, 0.04)	0.02	0.24	0.807
	EAT—total score	−0.002 (−0.04, 0.03)	0.02	−0.13	0.893
	**HQ—total score**	**0.08 (0.03, 0.12)**	**0.02**	**3.11**	**0.002** ^**^
	BFI-10—Openness	−0.03 (−0.06, 0.00)	0.02	−1.81	0.07
	BFI-10—Conscientiousness	0.003 (−0.03, 0.04)	0.02	0.17	0.864
	BFI-10—Extroversion	0.03 (0.00, 0.07)	0.02	1.72	0.086
	BFI-10—Agreeableness	−0.02 (−0.05, 0.01)	0.02	−1.44	0.150
	BFI-10—Emotional Stability	−0.01 (−0.05, 0.02)	0.02	−0.65	0.517
Physical health and Quality of life	**WHOQoL—total score**	**−0.04 (−0.08, 0.00)**	**0.02**	**−2.02**	0.043 *
School	**DEMS—Self-Efficacy**	**0.04 (0.00, 0.08)**	**0.02**	**2.16**	**0.031** ^*^
	DEMS—Family Engagement	−0.003 (−0.05, 0.04)	0.02	−0.13	0.895
	**DEMS—Aspiration**	**0.07 (0.04, 0.10)**	**0.02**	**4.14**	**<0.001** ^***^
	**DEMS—Attitudes**	**0.51 (0.47, 0.55)**	**0.02**	**26.54**	**<0.001** ^***^
	DEMS—Family Supervision	−0.02 (−0.06, 0.02)	0.02	−1.05	0.292
Family	**PBI—Care**	**0.06 (0.02, 0.11)**	**0.02**	**2.63**	**0.009** ^**^
	**PBI—Overprotection**	**−0.5 (−0.08, −0.01)**	**0.02**	**−2.77**	**0.006** ^**^

Standardized Beta effects are reported and grouped at the factor-level. In bold, significant variables are reported. ^*^p < 0.05, ^**^p < 0.01, ^***^p < 0.001.

## 4 Discussion

The present study aimed to investigate the socio-psychological factors contributing to dropout intention by assessing mental health indicators in a large Italian sample of high-school students. To achieve this goal, we conducted a multiple linear regression model including all predictors to estimate the association between factors and dropout intention. It is worth noting that, since predictors might be interrelated, especially in the domain of mental health, multicollinearity among factors was evaluated using Variance Inflation Factors (VIFs), resulting in acceptable levels of collinearity. Hence, the estimates of the regression model can be considered stable and not biased by overlapping constructs.

Overall, the results of the present study revealed a complex interaction between internal and external factors in predicting dropout intention. Indeed, psychological and contextual factors (e.g., quality of life, school-related factors, and family-related factors) play an important role in dropout intention within a comprehensive multivariate framework, with some acting as risk factors and others as protective factors. For example, QoL was negatively associated with dropout intention and could therefore be considered a protective factor, whereas specific DEMS factors, such as Attitudes, Aspiration, and Self-efficacy, were positively associated with dropout intention. Among psychological factors, only selected YSR subscales were significantly associated with dropout intention, with Attention Problems and Somatic Complaints showing positive associations while Aggressive Behavior and Thought Problems were negatively associated. Other behavioral domains, including eating attitudes (EAT), social media use (BSMAS), and internet gaming (IGDS), were not significantly associated with dropout intention when considered within the full model, with the only exception of the social withdrawal (HQ), that showed significant positive associations. Interestingly, the two PBI subscales on family care and overprotection displayed opposite directions of association with the intention to drop: “Overprotection” was negatively associated with dropout intention, while “Care” showed a positive association, suggesting differential roles of parental styles in dropout risk.

Moreover, our findings revealed that 24% of the adolescents surveyed exhibited risk of experiencing mental health issues. This is notably higher than the prevalence reported in prior studies, which have estimated that 10–20% of individuals encounter mental health difficulties during adolescence (European Commission, 2013). A potential explanation for this elevated risk could be linked to the impact of the COVID-19 pandemic. The restrictions and social disruptions associated with the pandemic have had detrimental effects on mental health, particularly in younger populations. Thus, these pandemic-related stressors may account for the worrisome increase in mental health risks observed in our data, compared to pre-pandemic levels (Solomou et al., 2024). The European Commission has furthermore identified psychological distress as a key factor contributing to early school leaving (European Commission, 2013). Our results align with this identification, highlighting the outstanding impact of attention disorders. Among all the indices of psychological distress, we found that the Attention Problems scale from the Youth Self-Report had the highest standardized beta, suggesting it as a highly potential predictive factor for school dropout. Attention Problems encompass a range of difficulties, such as acting immature for one’s own age, being unable to concentrate or maintain attention for long periods, and having trouble sitting still (Achenbach and Dumenci, 2001). These issues also include feeling confused or spaced out, daydreaming excessively, and acting without thinking. These behaviors can severely impact academic performance by making it challenging for students to follow lessons, complete assignments on time, and maintain consistent study habits (Barkley, 2015). Consequently, students exhibiting higher levels of Attention Problems often fall behind in their studies, leading to increased frustration and, ultimately, a higher likelihood of dropping out.

However, each of the measured mental health indices plays a critical role in the possible prediction of dropout intentions, coherently with the literature that suggests that the mere consequences of struggling with mental health represent a risk for school dropout (Kessler et al., 1997). Addressing these issues requires a comprehensive approach that considers the diverse ways in which mental health can affect educational outcomes. It should be emphasized that many mental disorders, including mood, anxiety, and personality disorders, do make their appearance in the second decade of life in over 60% of the cases, thus making adolescence the most critical period for vulnerability to mental health disruption (Jones, 2013). To make things even worse, these disorders often remain non-diagnosed and thus not treated, for a very long time, with dramatic consequences for the individual’s well-being, quality of life, and also for their school and professional choices (Solmi et al., 2021). By understanding and intervening in these areas, educators and policymakers can better support students at risk of dropping out and promote a more inclusive and supportive educational environment.

The results of the present study sharply highlight the family’s central role in ensuring the student’s academic achievement. Interestingly, the negative relation between the overprotection scale of the PBI questionnaire and the dropout intention suggest that the former might be a protective factor against dropout intention. This subscale measures the degree to which parents exert excessive control over their children, limiting their autonomy and promoting constant dependence. This can manifest in behaviors such as insisting on always knowing the child’s whereabouts and making decisions on their behalf. Overall, our results indicate that a highly protective parenting style may help against school dropouts for several reasons. Firstly, highly protective parents tend to monitor their children’s academic progress, ensuring that any academic or behavioral issues are promptly addressed, thereby reducing the risk of dropout (McNeal, 1999). Secondly, these parents are often deeply involved in their children’s lives, providing substantial emotional and practical support, which can help children overcome educational challenges (Pong and Ju, 2000). Lastly, overprotective parents are usually vigilant about risky behaviors, such as drug use or associating with negative peer groups (Romero and Ruiz, 2007), which can lead to school dropout. Their attentiveness helps prevent these behaviors, contributing to the child’s continued engagement in school. Overprotection has, however, its downsides, such as limiting children’s autonomy and ability to make independent decisions and to grow and learn to assume mature responsibility. Importantly, previous research highlighted that overprotective parenting style might entail long-term costs, such as reduced autonomy of the children, lower self-efficacy, and increased vulnerability to anxiety (Segrin et al., 2012; Spada et al., 2012). Thus, while parental supervision can protect against dropping out of school, it is essential to find a balance not to compromise children’s personal development and self-confidence (Bruysters and Pilkington, 2023).

Finally, we detected the impact of several demographic factors. Coherently with the literature, our data show a substantial disproportion regarding dropout intention across genders: young males are at a significantly higher risk of early school leaving than young females. Among many reasons, this asymmetry might reflect the current situation of the Italian job market, which still advantages men over women in finding employment at an earlier age (Borgna and Struffolino, 2017). Moreover, on average, girls showed a higher conscientiousness score, a personality trait associated with diligence and good school performance.

Another finding that emerged from our study concerns individuals who do not identify with a binary gender or who prefer not to declare their gender. This sub-sample reported the highest scores for dropout intention and significant psychological distress. This result aligns with literature showing that lesbian, gay, bisexual, and transgender (LGBTQ) youth face extreme discrimination within school environments and are at high risk for declining school performance, harassment, and social alienation (Bidell, 2014). Confronted with alienation and negative messages, their identity formation is often a difficult process. In such contexts, social support from peers and non-family adults, particularly from those who also identify as LGBTQ, has been shown to provide crucial emotional and informational support (Munoz-Plaza et al., 2002). As these issues appear to be rising steadily, research must focus on interventions, including fostering supportive peer networks and providing multiple resources within schools, to ensure academic and subjective well-being for these individuals.

Furthermore, over the last couple of decades, Italian schools have reflected the changes within Italian society, with an increasingly greater cultural and ethnic diversity, an impact detected by our data as well: 308 participants declared a mother tongue different than Italian, 189 were born in a different country, and 443 were born within Italian borders to at least one non-Italian parent. All these subsamples reported a dropout intention score higher than their Italian peers. The finding that students with a migration background (including first and second generations) reported significantly higher dropout intention warrants deeper reflection. Our data indicate that these students experience a distinct and often more challenging educational reality. While their academic attitudes are comparable to or even more positive than their Italian peers, they operate under considerable cumulative disadvantage. They report a lower overall quality of life, a lower socioeconomic status, and critically, a lower sense of academic self-efficacy coupled with lower educational aspirations, two factors strongly linked to dropout risk. Their social experience is also marked by greater isolation and a lower perceived quality of social life. This pattern aligns with broader research on the integration of migrant youth in Italy (Bianchi et al., 2021), which highlights how students from disadvantaged migrant backgrounds often face marginalization. Crucially, this same body of research suggests that peer acceptance and support within the classroom can act as a powerful protective factor, bolstering motivation and self-esteem. Our results, therefore, not only highlight a specific vulnerability but also point towards a promising avenue for prevention: targeted interventions that foster inclusive peer relationships and strengthen social support networks within schools could be key in mitigating the heightened dropout risk for these students. In addition, our results showed that a lower socioeconomic status not only characterizes these at-risk categories but also strongly correlates with the intention to abandon studies per se. In particular, lower socioeconomic status is associated with lower academic aspirations. Many young people belonging to lower social classes feel that high schools and universities are not suitable environments for them (Barone, 2006; Checchi and Flabbi, 2006). This aspect appears to impact at a psychological level even more than at a material one, as these people perceive a cultural and social distance that makes it difficult to imagine a future in a context that significantly breaks apart from their parental one.

The scope of the present research was mainly epidemiological, with a strong focus on the causes and determinants of school dropout. Nonetheless, the results may suggest some practical information regarding intervention implementation. First, it is extremely crucial to offer psychological counseling in the form of school psychologists, and/or develop training aimed at supporting teachers and professors in the task of identifying potential symptoms of attention deficit disorders. This approach allows for timely intervention, directing students to the appropriate services and preventing the worsening of psychopathological difficulties. An unequivocal evidence that emerges from research on the onset of psychopathological disorders is indeed the decisive role of the beginning of the therapeutic path: the sooner a disorder is recognized and treated (Dhejne et al., 2016), the greater the chances of resolving it, and the less time it will take to regain that mental well-being that is essential both for academic success and for a long and satisfying life. At the same time, it is important to implement policies that ensure equal opportunities, taking into account the specific needs of students with social vulnerabilities related to gender, geographical origin, or socioeconomic status. Fostering an empathetic and inclusive school environment not only helps the integration of marginalized groups but also creates a safer social network in which the individual can ask for help. Teachers, adequately trained, can then learn to recognize observable signs related to poor family participation or psychosocial difficulties, providing a first level of support and directing any critical cases towards more specialized resources.

Furthermore, the role of the internet and online connection cannot be overlooked. Adolescents represent the demographics that spend the most time online, especially on social media (Loe and Feldman, 2007; Vogels et al., 2022). Although intensive use of such platforms can lead to addictions, they also offer opportunities for social support and intimate disclosures (Yau and Reich, 2018). Therefore, the same platforms could be used for online-based interventions. Most of the existing knowledge on mental health promotion can thus be integrated into supportive interventions that might translate into digital delivery systems, or even take advantage of some of the benefits of online activities, such as peer-to-peer support.

Moreover, increasing awareness among parents and educators about the psychological, social, and contextual variables that contribute to school dropout can lead to a significant shift in mindset. When adults are informed about how factors affect school participation, they are more likely to adopt proactive, supportive attitudes. This change in mindset fosters a culture of shared responsibility, reduces stigma, and enhances the likelihood that students will receive the help they need in a timely and effective manner. Investing in psychoeducational initiatives aimed at families and teachers is therefore not only beneficial for students, but essential for building a more resilient and inclusive educational community (Simon, 2004). Consequently, building a school environment that favors collaboration between families, school, institutions, and psychological services can help ensure integrated support, thus promoting school participation and reducing the risk of dropping out.

### 4.1 Limitations

While the present study provides valuable insights into the predictors of school dropout, it is important to acknowledge a few limitations. The first significant limitation is the use of cross-sectional data. While this design allowed us to identify correlations between several personal and environmental factors, including mental health status and dropout risk, it does not establish causality. Indeed, the cross-sectional design does not allow us to determine which of the tested students actually dropped out of school, thereby enacting the intention measured by our questionnaires’ battery. Indeed, our study focused on dropout intention, which may nevertheless remain at the level of intention and not translate into actual behavior. Future studies adopting a longitudinal design are needed to extend and further validate the findings of the present study. Additionally, our dropout measure was based on self-reported intentions rather than actual dropout rates, which could be assessed only by a longitudinal design over several years. A further limitation is the presence of collinearity among the mental health measures. This methodological limitation reflects a fundamental feature of mental health, where everything is connected. Emotional, cognitive, and behavioral difficulties rarely occur in isolation; instead, they tend to cluster and reinforce one another in complex ways. Indeed, many of the indices were highly correlated with one another, making it challenging to disentangle the unique contributions of each mental health issue to dropout risk. As a result, it can be difficult to determine the specific impact of each measure independently, potentially obscuring nuanced relationships and interactions between different mental health factors. In addition to the collinearity due to the relationship between behavioral and psychosocial domains, self-report measures may have introduced common method variance, potentially inflating the observed associations between variables. Finally, for our investigation, we only recruited public schools, excluding students from private institutions, a choice that might limit the generalizability of our results. However, only 5% of students in Italian secondary schools go to a private institution (Ministero dell’Istruzione e del Merito, 2023), representing a minority of the Italian population of students. Furthermore, unlike public institutions, private schools are not free, with an average cost of about 7,000 euros per year (Nota di redazione, 2023) that inevitably generates a selection bias based on socioeconomic status. Finally, it is important, for future studies, to investigate and address the differences in the determinants of school dropouts between private and public schools. In summary, while our findings contribute to the understanding of dropout predictors, future research employing longitudinal designs and addressing collinearity issues will be crucial for elucidating and dissecting these relationships.

## 5 Conclusion

This study examined the socio-psychological determinants of school dropout intention in a large sample of Italian high-school students to investigate how individual and environmental factors jointly contribute to school dropout. Using standardized self-report measures and a series of linear regression models, we assessed the role of mental health indicators, personality traits, family functioning, and social conditions. The findings show that dropout intention is strongly associated with psychological distress, with attention-related difficulties emerging as the most plausible risk factor, while family supervision represents a potential protective element. Moreover, elevated dropout intention among males, students with a migration background, those from lower socioeconomic strata, and adolescents who do not identify within the gender binary highlights the impact of cumulative social disadvantage. Overall, the study supports the conclusion that school dropout intention reflects a multifaceted process in which psychological vulnerability and social inequality intersect, underscoring the need for integrated prevention strategies that combine mental health support, family involvement, and inclusive educational policies.

## Data Availability

The datasets presented in this study can be found in online repositories. The names of the repository/repositories and accession number(s) can be found at: https://osf.io/rqw4b/?view_only=600ea7e86f17424e9cb9743b9e28e724.
