# Supplementary material for: Socio-psychological predictors of school dropout intention among Italian adolescents: evidence from a large-scale study
Source: Front Psychol. 2026 Jun 1;17:1705896. doi: 10.3389/fpsyg.2026.1705896 (PMC13267494; doi:10.3389/fpsyg.2026.1705896)
Supplement: Supplementary file 1 [file Data_Sheet_1.PDF]

Supplementary materials

1. Multicollinearity check

Here, we report the results of the multicollinearity diagnostics for the full model. Variance Inflation Factors (VIFs) were computed for all predictors, and all values fell well below commonly accepted thresholds (i.e., VIF < 5), indicating low to moderate correlations among variables and no evidence of problematic multicollinearity, thereby supporting the stability and interpretability of the regression estimates. The analysis was conducted in Rstudio, through the *performance* package

```
> check_collinearity(full_model)
# Check for Multicollinearity
```

Low Correlation

Term	VIF	VIF 95% CI	Increased SE	Tolerance	Tolerance 95% CI
Gender	1.68	[1.59, 1.77]	1.29	0.60	[0.57, 0.63]
Age	1.15	[1.11, 1.21]	1.07	0.87	[0.83, 0.90]
SES	1.33	[1.28, 1.40]	1.16	0.75	[0.71, 0.78]
SocDesirability	1.78	[1.69, 1.88]	1.33	0.56	[0.53, 0.59]
Attention_YSR	2.71	[2.55, 2.88]	1.64	0.37	[0.35, 0.39]
RuleBreak_YSR	2.73	[2.57, 2.91]	1.65	0.37	[0.34, 0.39]
Aggressive_YSR	3.08	[2.90, 3.28]	1.76	0.32	[0.30, 0.35]
Withdrawn_YSR	3.74	[3.51, 4.00]	1.93	0.27	[0.25, 0.28]
Anxious_YSR	3.98	[3.73, 4.26]	2.00	0.25	[0.23, 0.27]
Thought_YSR	3.19	[2.99, 3.40]	1.78	0.31	[0.29, 0.33]
Social_YSR	3.15	[2.96, 3.35]	1.77	0.32	[0.30, 0.34]
Somatic_YSR	2.16	[2.04, 2.29]	1.47	0.46	[0.44, 0.49]
Other_YSR	2.07	[1.96, 2.19]	1.44	0.48	[0.46, 0.51]
BSMAS	1.47	[1.40, 1.55]	1.21	0.68	[0.65, 0.71]
IGDS	1.46	[1.39, 1.54]	1.21	0.69	[0.65, 0.72]
EAT	1.38	[1.32, 1.46]	1.18	0.72	[0.69, 0.76]
Total_HQ	2.98	[2.80, 3.18]	1.73	0.34	[0.31, 0.36]
Openness	1.13	[1.09, 1.19]	1.07	0.88	[0.84, 0.91]
Conscientiousness	1.53	[1.46, 1.62]	1.24	0.65	[0.62, 0.68]
Extroversion	1.97	[1.87, 2.09]	1.40	0.51	[0.48, 0.54]
Agreeableness	1.25	[1.20, 1.32]	1.12	0.80	[0.76, 0.83]
EmStability	1.72	[1.64, 1.82]	1.31	0.58	[0.55, 0.61]

QoL	2.02	[1.91, 2.14]	1.42	0.49	[0.47, 0.52]
SelfEfficacy_DEMS	1.69	[1.61, 1.78]	1.30	0.59	[0.56, 0.62]
FAM_Engag_DEMS	2.55	[2.40, 2.71]	1.60	0.39	[0.37, 0.42]
Aspiration_DEMS	1.27	[1.22, 1.33]	1.13	0.79	[0.75, 0.82]
Attitudes_DEMS	1.84	[1.74, 1.94]	1.36	0.54	[0.51, 0.57]
FAMSuperv_DEMS	1.82	[1.73, 1.93]	1.35	0.55	[0.52, 0.58]
Care_PBI	2.75	[2.58, 2.92]	1.66	0.36	[0.34, 0.39]
Overprotection_PBI	1.51	[1.44, 1.59]	1.23	0.66	[0.63, 0.69]
